# Comparative Transcriptomics Suggests Early Modifications by Vintec^®^ in Grapevine Trunk of Hormonal Signaling and Secondary Metabolism Biosynthesis in Response to *Phaeomoniella chlamydospora* and *Phaeoacremonium minimum*

**DOI:** 10.3389/fmicb.2022.898356

**Published:** 2022-05-17

**Authors:** Ana Romeo-Oliván, Justine Chervin, Coralie Breton, Thierry Lagravère, Jean Daydé, Bernard Dumas, Alban Jacques

**Affiliations:** ^1^Unité de Recherche Physiologie, Pathologie, et Génétique Végétales (PPGV), INP PURPAN, Université de Toulouse, Toulouse, France; ^2^Laboratoire de Recherche en Sciences Végétales, CNRS, UPS, Toulouse INP, Université de Toulouse, Toulouse, France; ^3^Metatoul-AgromiX Platform, MetaboHUB, National Infrastructure for Metabolomics and Fluxomics, LRSV, CNRS, UPS, Toulouse INP, Université de Toulouse, Toulouse, France; ^4^MetaboHUB-MetaToul, National Infrastructure of Metabolomics and Fluxomics, Toulouse, France

**Keywords:** biocontrol, *Trichoderma atroviride* SC1, *Phaeomoniella chlamydospora*, *Phaeoacremonium minimum*, esca, grapevine trunk diseases

## Abstract

Given their well-known antifungal abilities, species of the genus *Trichoderma* are of significant interest in modern agriculture. Recent studies have shown that *Trichoderma* species can induce plant resistance against different phytopathogens. To further extend this line of investigation, we investigate herein the transcriptomic response of grapevine trunk to Vintec^®^, which is a *Trichoderma atroviride* SC1-based commercial formulation for biological control of grapevine trunk diseases and which reduces wood colonization. The aim of the study is to understand whether the biocontrol agent Vintec^®^ modifies the trunk response to *Phaeoacremonium minimum* and *Phaeomoniella chlamydospora*, which are two esca-associated fungal pathogens. An analysis of transcriptional regulation identifies clusters of co-regulated genes whose transcriptomic reprogramming in response to infection depends on the absence or presence of Vintec^®^. On one hand, the results show that Vintec^®^ differentially modulates the expression of putative genes involved in hormonal signaling, especially those involved in auxin signaling. On the other hand, most significant gene expression modifications occur among secondary-metabolism-related genes, especially regarding phenylpropanoid metabolism and stilbene biosynthesis. Taken together, these results suggest that the biocontrol agent Vintec^®^ induces wood responses that counteract disease development.

## Introduction

Crops worldwide face multiple diseases that cause huge economic loss (Savary et al., 2019). Over the past decades, pest management has relied mostly on chemical control, which has caused serious harm to environmental and human health. An alternative to these conventional phytosanitary products is the use of living organisms or natural substances; a strategy known as biological control (Eilenberg et al., 2001). Although biological control offers numerous advantages over chemical pesticides, its main advantages are that it is environmentally friendly, cost-effective, and human safe technology (Cook, 1988; Bhat et al., 2013; Brodeur et al., 2018; Ram et al., 2018).

The main approach to managing plant disease *via* biological control consists in using antagonistic fungi or bacteria to combat plant pathogens (Thambugala et al., 2020). In this regard, the genus *Trichoderma* is of great interest (Vinale et al., 2008; Zin and Badaluddin, 2020; Ferreira and Musumeci, 2021). *Trichoderma* is a genus of fungi inhabiting a large spectrum of soils and its species are opportunistic plant symbionts (Vinale et al., 2008) that attack or compete with other fungi by using various direct mechanisms, such as the production of antimicrobial compounds or lytic enzymes or the competition for resources (Kubicek et al., 2001; Benítez et al., 2004). To date, 25 *Trichoderma* species have been identified as potential biological control agents (BCAs) and several commercial formulations based on different strains of *Trichoderma* are available (Ferreira and Musumeci, 2021).

*Trichoderma* spp. also act indirectly by reprogramming gene expression in the host (Shaw et al., 2016). The associations between *Trichoderma* spp. and the host plant often improve plant resistance to disease, augment abiotic stress tolerance, and enhance other developmental processes. For instance, Zhao et al. (2021) reported increased growth of tomato plants upon inoculation by *T. afroharzianum*. In addition, *T. longibrachiatum* reportedly enhances the salt tolerance of wheat (Zhang et al., 2016) and the water-stress response on tomato (De Palma et al., 2019). *T. harzianum* enhances the antioxidant response (Mastouri et al., 2012) and also stimulates the production of jasmonic acid and salicylic acid, which reduces the incidence of root nematodes on tomato seedlings (Yan et al., 2021).

In viticulture, biological control has attracted significant interest, especially against pathogens associated with grapevine trunk diseases (GTDs). GTDs are a growing concern because of their increasing incidence in vineyards worldwide, which reduces yield and productivity, degrading significantly the economics of the viticulture industry (Fontaine et al., 2016; Mondello et al., 2018a,b). Esca, Botryosphaeria dieback, and Eutypa dieback are currently the three main GTDs in European vineyards. Esca is a complex disease with symptoms that vary depending on the age of the vine and the infecting pathogen (Mugnai et al., 1999; Graniti et al., 2000; Bertsch et al., 2013). Numerous pathogenic agents are associated with Esca; the principal causing agents are *Phaeomoniella chlamydospora*, *Phaeoacremonium minimum* (previously *Phaeoacremonium aleophylum*) (Larignon et al., 2000), *Fomitiporia mediterranea*, and other *Phaeoacremonium* spp. (Gramaje et al., 2015). Since the ban of sodium arsenate and benzimidazoles in the early 2000s, no effective treatment has been found to eradicate these diseases. Instead, an integrated pest-management strategy has been established that consists of different prophylactic treatments both in nurseries and in vineyards (Mondello et al., 2018a,b).

In recent years, the use of various strains of *Trichoderma* has produced good results against pathogens associated with these diseases. In grapevine nurseries, the application of *T. harzianum* during propagation proved as effective in combating these diseases as the use of conventional phytosanitary products (Fourie et al., 2001). Similar results were obtained by Di Marco et al. (2004); Di Marco and Osti (2007), who reported a reduced incidence of esca-associated pathogens and improved morphological features. In vineyards, the application of various *Trichoderma* spp. for protecting pruning wounds reduced the presence of pathogens (Kotze et al., 2011), decreasing the percentage of infected plants and reducing the severity of symptoms (Bigot et al., 2020).

In France, three *Trichoderma*-based biocontrol commercial solutions certified against GTDs are currently available. One is a combination of *T. asperellum* ICC012 and *T. gamsi* ICC080 (Escalator^®^, De Sangosse); the other two are formulations of *T. atroviride* I1237 (Esquive WP^®^, Agrauxine by Lesaffre) and *T. atroviride* SC1 (Vintec^®^, Belchim Crop Protection). Recent studies designed to evaluate the efficacy of Vintec^®^ showed promising results in the battle against GTDs. Pertot et al. (2016) reported that the application of the BCA Vintec^®^ at different stages of the propagation process decreases vine infections by *P. minimum* and *P. chlamydospora*. In addition, Berbegal et al. (2020) confirmed that the application of the BCA Vintec^®^ in the nursery reduces the incidence of diverse GTDs and showed that subsequent treatments in vineyards provide long-term protection against GTDs.

The results of a previous study show that the application of the BCA Vintec^®^ on grapevine wood triggers significant metabolic changes upon infection with *P. chlamydospora* and *P. minimum*. Three weeks after infection, the different classes of stilbenoid and flavonoid compounds, known for their antifungal properties, are differently produced in plants pre-inoculated with Vintec^®^ compared with non-pre-inoculated plants (Chervin et al., 2022). In the present work, we investigate rapid transcriptomic reprogramming in grapevine trunk in response to inoculation with the BCA Vintec^®^, which is a commercial *T. atroviride* SC1-based solution. The aims of this study are thus (i) to investigate the plant molecular mechanisms modified by the presence of the BCA Vintec^®^ and (ii) to determine whether the Vintec^®^ treatment enhances plant response against the two esca-associated pathogens *P. chlamydospora* and *P. minimum*.

## Materials and Methods

### Fungal Material

This study used the fungal strains *P. minimum* CBS 100398 and *P. chlamydospora* CBS 239.74 from the Westerdijk Fungal Biodiversity Institute (Utrecht, Netherlands). Both fungi were grown in Malt Extract-Agar (MEA) at 26°C in the dark. The BCA Vintec^®^ (*T. atroviride* SC1, 2 × 10^10^ conidia per gram of formulated product) was provided by Belchim Crop Protection, and the Vintec^®^ suspension was prepared at 2 g/L in autoclaved distilled water.

### Plant Material

One-year-old canes of Cabernet Sauvignon clone 15 were collected from Daydé Nurseries (Pépinières Daydé, Montans, Occitanie, France) in January 2019 and divided into two-node dormant cuttings. The cuttings were surface-disinfected in a 10 L bath of 0.05% bleach (2.6% active chloride) for 30 s, then in a 10 L bath of 0.05% 8-hydroxyquinoline sulfate (Beltanol^®^, Syngenta) for 12 h in a cold chamber (4°C). After the bleach disinfection, the cuttings were rinsed twice with clear water, and after the 8-hydroxyquinoline sulfate disinfection, the cuttings were rinsed three times with clear water. Cuttings were first planted in autoclaved (121°C, 15 min) mineral wool to allow rooting and budding. Three weeks after the emergence of the first leaf, cuttings were transferred to individual pots filled with potting substrate (PAM2, PROVEEN Substrates). The plants were then grown in plant growth tents (photoperiod 12 h/12 h, 45% humidity, 25°C).

### Plant Inoculation

The plants were drilled at the internode and inoculated with 20 μL of Vintec^®^ suspension or 20 μL of sterile water. Five days after Vintec^®^ inoculation, the plants were infected with two plugs of colonized agar, one of each fungus. The injuries were protected with parafilm (American National Can, Chicago, United States). After the infection, five different conditions were studied: non-injured/non-infected plants (NINi, *n* = 25), injured/non-infected plants (INi, *n* = 25), inoculated with Vintec^®^ (IV, *n* = 25), infected with both fungi (IPP, *n* = 25), and inoculated with Vintec^®^ and infected with both fungi (IVPP, *n* = 25). Wood samples of 1 cm above and below the inoculation site were taken at 48 h post-infection (hpi) and 6 weeks post-infection (wpi). Four biological replicates consisting of a pool of four plants were collected for each condition at 48 hpi. At the latest time (i.e., 6 wpi), only three pools of three plants were taken. Immediately after collection, the samples were frozen in liquid nitrogen and stored at –80°C. Before RNA and DNA extraction, the samples were lyophilized and ground. The samples collected 6 wpi were used to confirm pathogen infection *via* qPCR quantification of the Pm/Pch β-tubulin gene as per Pouzoulet et al. (2013).

### RNA Extraction, DNAse Treatment, and Reverse Transcription

One milliliter of RNA extraction buffer (CTAB 2%, PVPP 2%, Tris 300 mM, EDTA 25 mM, NaCl 2 M, pH 8, 2% β-mercaptoethanol) was added to one hundred milligrams of wood powder and the mix was incubated 10 min at 65°C under continuous agitation (9,000 rpm). The samples were then centrifuged for 15 min at 10,000 rpm, 4°C. One volume of chloroform: isoamyl alcohol (24/1, v/v) solution was added to the supernatant, following which the solution was centrifuged for 15 min at 10,000 rpm, 4°C to form two phases. The upper aqueous phase contains nucleic acids. This stage was repeated twice.

To allow the nucleic acids to precipitate, 0.6 volume of isopropanol and 0.1 volume of CH_3_COONa 3M were added to the upper phase, following which the samples were stored overnight at –80°C. The next day, the samples were centrifuged for 30 min at 10,000 rpm, 4°C. The resulting pellet was redissolved in 300 μL of SSTE buffer (NaCl 1 M, SDS 0.5%, Tris 10 mM, EDTA 1 mM, pH 7) and 600 μL of pure ethanol. The mixture was transferred to a mini spin column (RNeasy mini kit, Qiagen, United States). The successive steps were done as described in the kit handbook. RNA was eluted in 30 μL of RNase-free water, aliquoted in 6 μL samples, and stored at –80°C. RQ1 RNase-Free DNase kit (Promega, United States) was used to eliminate the possible co-purified genomic DNA.

### RNA Sequencing and Data Analysis

cDNA library preparation and sequencing were done by using the genomic platform GeT-Plage (GenoToul, INRA, Toulouse, France). The cDNA libraries were prepared with the help of TruSeq Stranded mRNA (Illumina) and then sequenced by using an Illumina HiSeq 3000 sequencer as paired-end reads of 150 bp (Illumina, CA, United States). Quality control was done by using fastqc (v0.11.9; Andrews, 2010). If necessary, quality trimming was done by using sickle-trim (v1.33; Joshi and Fass, 2011) or cutadapt (v2.10; Martin, 2011) to remove adapters. Mapping to the reference genome 12X (Canaguier et al., 2017) was done by using STAR (v2.7.5a; Dobin et al., 2013), and mapped counts were extracted by using featureCounts (subread v2.0.1; samtools v1.9; Liao et al., 2014). Differential expression analysis was performed using the R package SARTools^1^. All conditions were compared with the NINi control (i.e., INi vs NINi, IV vs NINi, IPP vs NINi, IVPP vs NINi) and differentially expressed genes (DEGs; *p*-value < 0.05) were extracted. DEGs induced by the injury (INi vs NINi) were discarded for the remainder of the analysis. DEG functional annotations were assessed by using the VitisNet functional annotation database (Grimplet et al., 2009). Clustering was analyzed by using R with the *pheatmap* package, and functional enrichment was calculated in R by using Fisher’s exact test.

### RT-qPCR

Five genes were tested by RT-qPCR to validate RNA seq results. Gene candidates (listed in Table 1) satisfied the following criteria: (i) they were differentially expressed compared with the control condition, (ii) they were specific to a single condition, and (iii) they served an interesting putative biological function. For RT-qPCR analysis, cDNA was first synthesized from RNA samples by reverse transcriptase reaction using the GoScript^®^ reverse transcriptase Kit (Promega, United States). RT-qPCR reactions were done in an ABI 7500 Real-Time PCR cycler (Applied Biosystems, Foster City, United States) using GoTaq RT-qPCR systems (Promega, United States) in a final volume of 10 μL. Primers were designed by using Primer Blast (Ye et al., 2012; Optimal Tm = 62°C, 50–60% GC) and were used at a concentration of 0.5 μM. The cycling program consisted of 95°C for 5 min (initial denaturation); 40 cycles of 15 s at 95°C (denaturation) followed by 45 s at 62°C (annealing and extension); and an additional melting analysis of 40 min from 60 to 95°C. The ABI SDS software v.1.4 (Applied Biosystems, Foster City, United States) was used to collect the amplification data. Gene expression was calculated by using the 2^–ddCt^ method (Zhang et al., 2020).

**TABLE 1:** List of primers for RT qPCR designed for validating the RNA sequencing analysis.

Gene ID	Primer	Sequence	Tm
VIT_04s0023g03010	Glyco Fw	5′-GGTCTCTCCTGCTCGGATGT-3′	61.0
	Glyco Rv	5′-ATTCAGGCACCAACCTGGGG-3′	62.0
VIT_12s0028g00690	AUXR Fw	5′-GAAGGTCCCTGTGCTCGTTG-3′	60.2
	AUXR Rv	5′-TGCTTGAACAGCTTTGTCGGT-3′	60.7
VIT_05s0049g00390	E8 Fw	5′-AGACGTGCGGTTGCCATTTC-3′	61.9
	E8 Rv	5′-GAGTGAGCCCAGCATCCAGT -3′	61.9
VIT_05s0077g01570	PATHO10 Fw	5′-CTCTGCAAACCAACCATTCCTCC-3′	61.9
	PATHO10 Rv	5′-CACTCTCGTAAGTGAAAACACCCA-3′	61.0
VIT_16s0100g00860	ChalcS Fw	5′-GGAAGCAGCATTGAAGGCCC-3′	61.9
	ChalcSRv	5′-GCAGTTTCTGCATAGCACCCT-3′	61.0

## Results

### Transcriptomic Woody Response to BCA Vintec^®^, Pathogens, and Wound

We performed RNA sequencing on wood samples inoculated with the BCA Vintec^®^ (IV), the two pathogens (IPP), or the BCA Vintec^®^ in combination with pathogens (IVPP). An injured control (INi) was added to the analysis to select DEGs affected only by the treatment and not by the injury. We obtained an average of 20.4 M reads per condition and pool. The percentage of reads aligned to the reference genome of *Vitis vinifera* (12X.0 version, cv PN40024) varies depending on the condition (see Supplementary Table 1). The transcriptome of reference (*Vitis_vinifera.12X.48.chr.gtf.gz*, from Ensembl plants genomes) was used for gene annotation. Differential analysis was applied to transcript normalized counts. All test conditions were compared with the non-injured/non-inoculated control (i.e., INi vs NINi; IPP vs NINi; IV vs NINi; and IVPP vs NINi).

**FIGURE 1:**
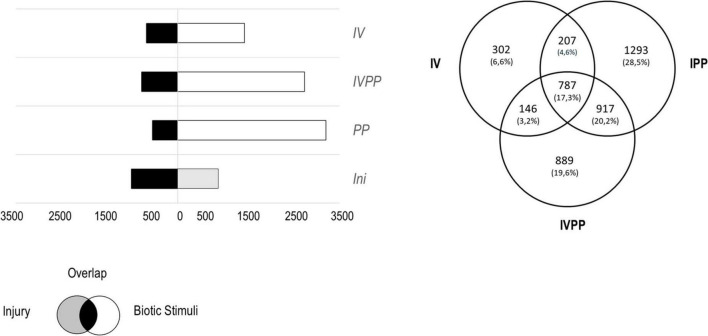
**(A)** Number of the total differentially expressed genes (DEGs, p-value < 0.05) by the injury, in the presence of Vintec^®^ (IV), during the infection (IPP) or during the infection in plants pre-inoculated with Vintec^®^ (IVPP). In black, DEGs induced by the injury and the biotic stimulus; in gray, DEGs induced by the injury alone; in white, DEGs induced by each of the three biological stimuli. **(B)** Venn diagram representing specific and common DEGs for all the three biotic stimuli.

The injury alone modified the expression of 1,869 genes (*p*-value < 0.05), whereas the number of DEGs induced by the BCA Vintec^®^ alone, the two pathogens, and the BCA Vintec^®^ in combination with the two pathogens was 2,114, 3,748, and 3,519, respectively. From these sets of DEGs, we discarded those that were in response to injury, which left 1,442 DEGs in response to the BCA Vintec^®^, 3204 DEGs in response to the two pathogens, and 2,739 DEGs in response to the two pathogens in the presence of the BCAVintec^®^ (Figure 1A). The pattern expression of five candidate genes was confirmed by qRT-PCR analysis (Supplementary Figures 1, 2) as validation of the RNAseq analysis.

The comparison of the three sets of DEGs revealed that only 787 genes were differentially expressed in all three conditions, which represents 17.3% of the total DEGs. The presence of Vintec^®^ specifically induced the differential expression of 448 genes, from which 146 genes were also differentially expressed in the presence of the two pathogens. Infection with the two pathogens modified the expression of 2,210 genes, of which 917 genes overlapped with infection with the two pathogens in the presence of the BCA Vintec^®^. Interestingly, the combination of the two pathogens with Vintec^®^ modifies the expression of 889 genes, which represents 19.6% of the total DEGs (Figure 1B). In other words, 889 genes were differentially expressed when the plants were inoculated with BCA Vintec^®^ prior to pathogen infection.

### Gene Clustering Leads to Identification of Three Groups of Co-regulated Genes

**FIGURE 2:**
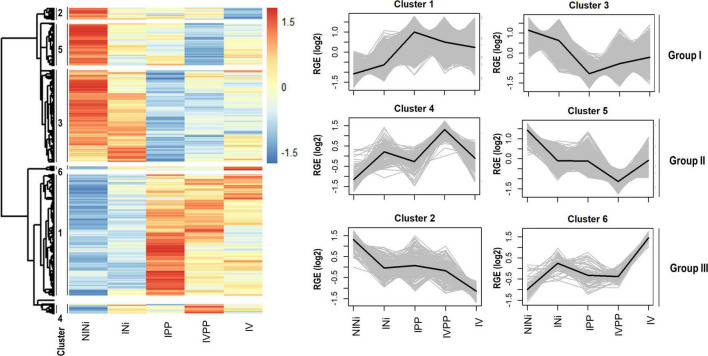
Cluster analysis of the total DEGs associated to the different biotic stimuli. The heatmap shows the different gene expression patterns. Each line represents one gene, and each column represents a biological condition. The color key indicates log2 relative gene expression (RGE) values. The numbers on the left of the heatmap indicated the different clusters. DEGs were arranged in six different clusters depending on their pattern profile. Dendrograms represent the hierarchical distance between genes and clusters. On the right, the expression pattern for each cluster is showed. Clusters were rearranged in three groups: Clusters 1 and 3 (Group I), responding to the three biotic stimuli but specially to the infection; clusters 2 and 6 (Group III), responding to the BCA Vintec^®^; and clusters 4 and 5 (Group II), responding to the infection and the BCA Vintec^®^.

For further analysis, the DEGs were separated into three groups based on their expression pattern (Figure 2). Each group was composed of two clusters of co-regulated genes showing patterns of expression. Group I contained genes whose expression was modified by one of the three biotic stimuli, but more strongly by the two pathogens (Cluster 1 and 3). Group II contained genes whose expression was modified by the presence of the two pathogens in plants pre-inoculated with the BCA Vintec^®^ (Clusters 4 and 5), and Group III contained genes whose expression was modified by the presence of the BCA Vintec^®^ (Clusters 6 and 2).

Group I contains the majority of DEGs (76.5%). The expression pattern of the DEGs in this group is thus very heterogeneous. Group III regroups the smallest portion of total DEGs (5%). Based on their expression pattern, the induction or repression of these genes seem to specifically respond to the presence of Vintec^®^. The rest of the DEGs (18.5%) appears in clusters 4 and 5 (Group II). The expression of these genes is up- or downregulated by the presence of the two pathogens in plants pre-inoculated with the BCA Vintec^®^. The greater percentages of annotated DEGs in the three groups (23.6, 18.0, and 20.5%, respectively) were associated with “Primary Metabolism” functions, followed by “Signaling” (7.6%) and “Regulation” (7.4%) in Group I, “Transport elements” (10.6%) and “Regulation” (8.8%) in Group II, and “Transport elements” (12%) and “Cellular process” (8.1%) in Group III.

### Functional Enrichment Gives Insights Into Different Perception of Vintec^®^ and Pathogen Infection

Functional enrichment analysis (Fisher’s exact test, *p*-value < 0.05) was applied to the three groups (see results in Tables 2–4). The “Glutathione metabolism” (*p*-value = 3.291 × 10^–10^), “Phenylpropanoid biosynthesis” (*p*-value = 2.481 × 10^–5^), and “Tyrosine metabolism” (*p*-value = 2.53 × 10^–4^) categories are enriched for upregulated DEGs in Group I. Among upregulated functions, “ABA biosynthesis” and “ethylene-mediated signaling pathway” are also enriched (*p*-value = 1.49 × 10^–3^ and 3 × 10^–3^, respectively), whereas “Cytokinin inactivation” (*p*-value = 4.92 × 10^–2^) joins the downregulated categories in Group I. No significant enrichment for categories associated with “Hormone signaling” occurs among DEG functions of Group II. In contrast, in Group III, auxin-mediated signaling appears to be repressed because auxin-responsive elements (VIT_03s0038g01130, VIT_03s0038g00950, VIT_19s0085g00010) are strongly downregulated compared with the NINi control, and auxin inactivation-related genes are upregulated.

**TABLE 2:** Functional enrichment in Group I. DEGs up- and down-regulated by the two pathogens (IPP).

Functional category	Regulation	n DEGs	*p*-value
Glutathione metabolism	UP	15	3.29 × 10^–10^
Phenylpropanoid biosynthesis	UP	12	2.48 × 10^–5^
Tyrosine metabolism	UP	5	2.53 × 10^–4^
Transcription factor: Zinc finger B-box family	UP	3	1.02 × 10^–3^
ABA biosynthesis	UP	2	1.49 × 10^–^_3_
Wounding	UP	3	1.59 × 10^–3^
Pentose glucuronate interconversion	UP	6	1.74 × 10^–3^
Photosynthesis, Photosystem II	UP	4	1.81 × 10^–3^
Flavonoid biosynthesis (Anthocyanin)	UP	6	2.00 × 10^–3^
Ethylene-mediated signaling pathway	UP	9	3.00 × 10^–3^
Cell wall organization and biogenesis	DOWN	7	6.12 × 10^–5^
Calcium sensors and signaling	DOWN	5	1.35 × 10^–4^
DNA metabolism	DOWN	4	7.73 × 10^–^4
Major Facilitator Superfamily. Anion:Cation Symporter	DOWN	1	1.50 × 10^–2^
Methionine metabolism	DOWN	2	2.26 × 10^–2^
Protein metabolism and modification: Protein folding	DOWN	3	2.30 × 10^–2^
Channels and pores: A-Type channels. CorA Metal Ion Transporter	DOWN	1	2.49 × 10^–2^
Photosynthesis, antenna proteins	DOWN	1	4.44 × 10^–2^
Cytokinin inactivation	DOWN	1	4.92 × 10^–2^

**TABLE 3:** Functional enrichment in Group II. DEGs up- and down-regulated by the two pathogens and Vintec^®^ (IVPP).

Functional category	Regulation	n DEGs	*p*-value
Phenylpropanoid biosynthesis	UP	5	2.57 × 10^–4^
Porters. Phosphate Permease (Pho1)	UP	2	9.18 × 10^–4^
Sphingolipid biosynthesis	UP	1	1.29 × 10^–2^
Glycolysis Gluconeogenesis	UP	3	1.65 × 10^–2^
Transcription factor: BSD family	UP	1	1.93 × 10^–2^
Aerolysin Channel-forming Toxin	UP	1	2.87 × 10^–2^
Biotic stress response	UP	3	3.04 × 10^–2^
Protein transport, Tethering factors	UP	2	3.38 × 10^–2^
Monoterpenoid metabolism	UP	2	3.38 × 10^–2^
Glycan catabolism	DOWN	1	3.27 × 10^–3^
Plastid outer envelope protein 16 kDa	DOWN	1	6.53 × 10^–3^
Regulation of translation (activation)	DOWN	1	6.53 × 10^–3^
DNA recombination and repair	DOWN	3	8.07 × 10^–3^
Drug/Metabolite Transporter	DOWN	3	1.16 × 10^–2^
Biotic stress response: Plant-pathogen interaction (R-prot)	DOWN	4	1.50 × 10^–2^
Pentatricopeptide domain family	DOWN	5	1.59 × 10^–2^
Fatty acid metabolism, Alpha-linolenic acid metabolism	DOWN	2	1.93 × 10^–2^
Ammonia Channel Transporter	DOWN	1	2.91 × 10^–2^

**TABLE 4:** Functional enrichment in Group III. DEGs up- and down-regulated by the BCA Vintec^®^.

Functional category	Regulation	n DEGs	*p*-value
Cell wall organization and biogenesis	UP	10	8.01 × 10^–4^
Channels and pores: beta-barrel porins	UP	3	3.05 × 10^–3^
Photosynthetic-chain phosphorylation	UP	3	3.52 × 10^–3^
Auxin inactivation	UP	2	7.81 × 10^–3^
Transcription factor: WRKY family	UP	3	8.03 × 10^–3^
Phenylalanine metabolism	UP	4	9.62 × 10^–3^
Porters. Oligopeptide Transporter	UP	2	1.26 × 10^–2^
Protein Kinase Signaling	UP	16	1.32 × 10^–2^
Abiotic stress response: UV	UP	1	1.48 × 10^–2^
Propanoate metabolism	UP	2	1.71 × 10^–2^
Glycerolipid biosynthesis	DOWN	1	6.75 × 10^–3^
Transcription factor: FHA-like family.	DOWN	1	1.05 × 10^–2^
Light signaling	DOWN	1	1.20 × 10^–2^
Auxin-mediated signaling pathway	DOWN	2	1.39 × 10^–2^
Protein folding	DOWN	2	1.40 × 10^–2^
G-protein signaling. RAB-GTPase regulation	DOWN	1	1.72 × 10^–2^
Senescence	DOWN	1	1.72 × 10^–2^
Transcription factor: Trihelix family	DOWN	1	2.09 × 10^–2^
Riboflavin metabolism	DOWN	1	2.45 × 10^–2^
Transcription factor: G2-like family.	DOWN	1	2.60 × 10^–2^

**FIGURE 3:**
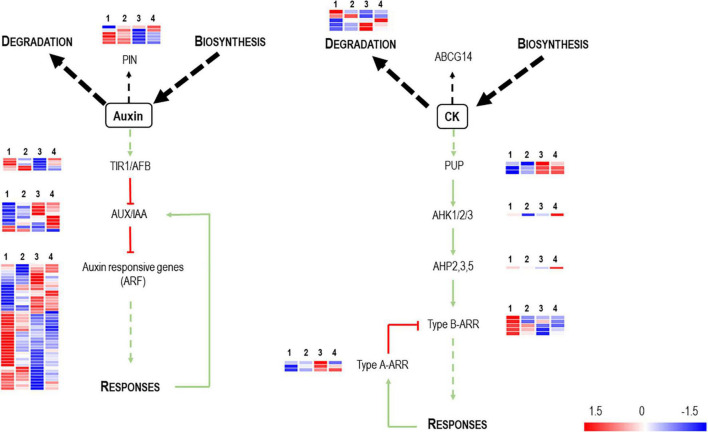
Schematic representation of auxin and cytokinin metabolism, transport and signaling pathways (adapted from Schaller et al., 2015). PIN and ABCG14 are efflux carriers which transport auxin and cytokinin, respectively, throughout the plant. Auxins binds TIR1 receptors with induces the degradation of the AUX/IAA complex, which are transcriptional repressors of the Auxin Response Factors (ARFs). This induces the relieve of the repression of the ARFs and thus the activation of the expression of auxin related genes. In the case of cytokinin signaling, three components are involved: cytokinin receptors, AHKs; their downstream targets, AHPs; which activates the ARRs. There exist two categories of ARR (types A and B). Type-B ARRs positively control the response to cytokinin and type-A ARRs negatively regulate cytokinin signaling. The heatmaps represent the expression profiles of the identified DEGs associated to these gene functions. The color key represents the log2 relative gene expression values, red for upregulated genes and blue for downregulated genes. Each line represents one gene, and each column represents one condition (1*^st^* column, NINi; 2*^nd^* column, INi; 3*^rd^* column, IVPP; 4*^th^* column, V).

Auxins, cytokinins, and gibberellins control plant vegetative development and growth by coordinating cell proliferation and differentiation (Vanstraelen and Benkov, 2012). No DEGs appear related to gibberellin signaling. However, in addition to auxins, differential regulation of genes is associated with cytokinin biosynthesis and signaling (Figure 3). The analysis of gene expression associated with “auxin-mediated signaling pathways” in the presence of Vintec^®^ reveals an inverse gene expression pattern compared with the NINi control. Most auxin-responsive putative genes (ARFs) are repressed by the presence of Vintec^®^, and this repression is more significant under the IPPV condition. In addition, auxin inactivation elements seem to be over-expressed in IV and IVPP. Altogether, these results suggest that auxin signaling pathways are repressed by the presence of Vintec^®^. These statements are also valid for cytokinin-mediated signaling pathways. Cytokinin type-A responsive elements, which negatively regulate type-B responsive elements, are induced in both IV and IVPP conditions, as well as cytokinin catabolic pathways. Furthermore, the category “cell wall organization and biogenesis” is enriched among upregulated genes of Group III (*p*-value = 8.01 × 10^–4^) but downregulated in Group I (*p*-value = 6.12 × 10^–5^), which could be linked to these differences in hormonal signaling.

### BCA Vintec^®^ Enhances Reprogramming of Secondary Metabolism Gene Expression During Pathogen Infection

**FIGURE 4:**
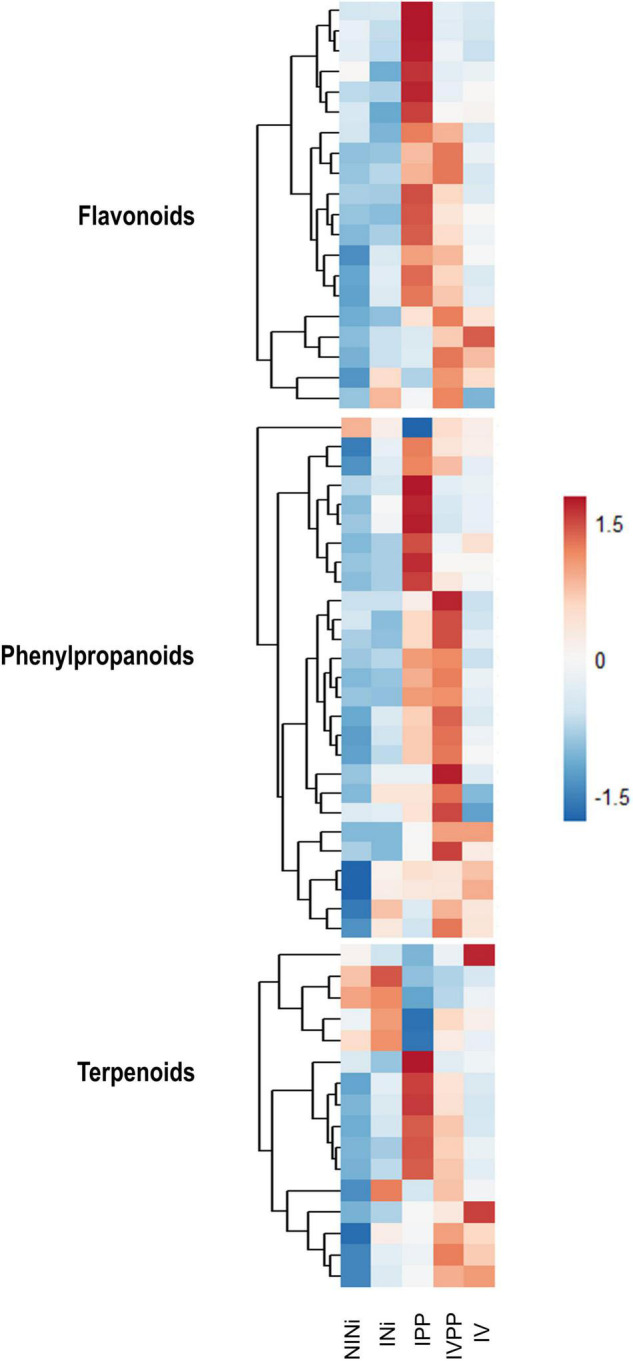
Heatmap representing the detected DEGs associated to flavonoids, phenylpropanoids and terpenoids biosynthesis. The color key represents the log2 relative gene expression values. Each line represents one gene, and each column represents one condition.

Among the up-regulated DEGs in Group II, the category “Phenylpropanoid biosynthesis” is significantly enriched (*p*-value = 2.57 × 10^–4^), along with “Monoterpenoid metabolism” (*p*-value = 3.38 × 10^–2^), which is also related to “Secondary metabolism.” Interestingly, when studying the expression pattern of DEGs associated with “Flavonoid biosynthesis,” “Terpenoid biosynthesis,” or “Phenylpropanoid biosynthesis,” we observe specific signatures (Figure 4). Some genes are up-regulated in the presence of the two pathogens, but their expression remains unchanged in plants pre-inoculated with Vintec^®^. In contrast, we observe another set of genes responding specifically to the infection only in the presence of Vintec^®^.

**FIGURE 5:**
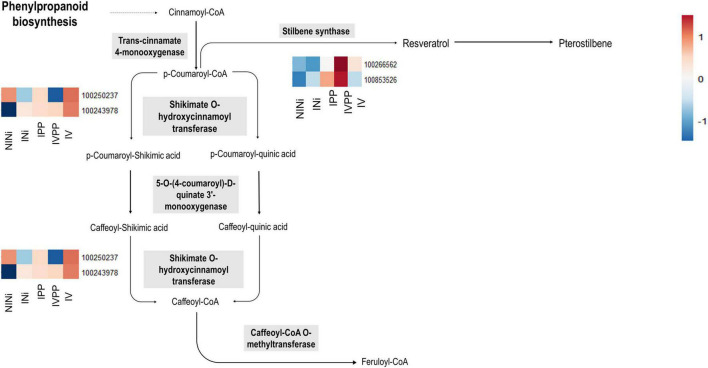
Schematic representation of stilbene biosynthesis (Adapted from KEGG Pathways). The heatmap represent the expression profile of the identified DEGs associated to the ENTREZ IDs of the enzymes implicated in this biosynthesis pathway. The color key represents the log2 relative gene expression values. Each line represents one gene, and each column represents one condition.

Secondary metabolites such as flavonoids, terpenoids, or stilbenes are known to play important roles in plant protection against biotic and abiotic stresses (Samanta et al., 2011). Interestingly, the upregulation of some of these genes is exclusive to the IVPP condition. The results show that genes VIT_10s0042g00920 and VIT_16s0100g00940 (ENTREZ ID 100266562 and 100853526, respectively) are strongly upregulated in plants inoculated with Vintec^®^ before infection. These genes code for two putative stilbene synthases, which are enzymes associated with the biosynthesis of resveratrol and pterostilbene. In contrast, VIT_110037g00440 (ENTREZ ID 100250337), another gene associated with this same metabolic pathway, is significantly repressed during the infection in the presence of the BCA. The first two genes code for the stilbene synthase, which is implicated in the conversion of coumaroyl-CoA into *trans*-resveratrol, whereas the third gene codes for a shikimate *O*-hydroxycinnamoyltransferase, which catalyzes the interconversion of coumaroyl-CoA into caffeoyl-CoA (Figure 5).

## Discussion

*Trichoderma* spp. have been known since the 1920s for their ability to compete with other fungi *via* direct mechanisms such as competition for nutrients and space, mycoparasitism, or antibiosis (Harman, 2006). However, the ability of *Trichoderma* spp. to trigger modifications in plant response was not studied until the 1990s (De Meyer et al., 1998). Since then, the way in which *Trichoderma* spp. affect plant species in response to diverse abiotic and biotic stimuli has been widely studied (Tucci et al., 2011; De Palma et al., 2019; Wang et al., 2020; Yan et al., 2021). For instance, several studies report that *Trichoderma* spp. modulates growth promotion while enhancing salt and water stress tolerance *via* plant transcriptome reprogramming in *Arabidopsis thaliana* (Brotman et al., 2013), tomato (Mastouri et al., 2012; De Palma et al., 2019; Zhao et al., 2021), wheat (Zhang et al., 2016), and cacao (Bae et al., 2009). In another example involving tomatoes, the application of *T. harzianum* helps activate plant defense reactions against root-knot nematodes and stink bugs (Alınç et al., 2021) by enhancing secondary metabolite production and the activation of defense-related enzymes (Yan et al., 2021). Defense induction by *Trichoderma* spp. is also reported in cucumber (Yedidia et al., 2003; Shoresh et al., 2005), *A. thaliana* (Mathys et al., 2012), rice, and cotton (Djonović et al., 2006). In the present work, we study the transcriptome reprogramming in grapevine trunk triggered by the commercial biocontrol solution Vintec^®^ (*Trichoderma atroviride* SC1) both alone and during infection with *P. chlamydospora* and *P. minimum*, the two main pathogens associated with esca disease.

Recent studies have shown that pathogen infection triggers the reprogramming of gene expression in grapevine trunk (Pierron et al., 2016; Massonnet et al., 2017; Gonçalves et al., 2019; Labois et al., 2020). Here, we confirm that the combined infection of *P. chlamydospora* and *P. minimum* leads to the modification of a large set of genes. The presence of the BCA Vintec^®^ alone also modifies gene expression of grapevine trunk but to a lesser extent than pathogen infection, suggesting that grapevine trunk perceives and differentially responds to the BCA or the pathogens. Functional enrichment analysis also shows dissimilarities between the BCA Vintec^®^ and the two pathogens.

Hormone signaling depends on the biotic stimuli. Plant hormones orchestrate plant development over different stages, are key actors in virtually every physiological plant process, and are vital for maintaining the balance between defense and growth when a plant is stressed (Pieterse et al., 2012; Naseem et al., 2015). Several studies show that the establishment of the plant-*Trichoderma* interaction and the ability to activate systemic resistance depends on different mechanisms employing jasmonic acid (JA) and ethylene (ET) signaling pathways (Shoresh et al., 2005, 2010; De Palma et al., 2019). The role of auxin signaling has been widely studied in connection with growth promotion induced by *Trichoderma.* Initially, *Trichoderma* was thought to modulate plant growth *via* the production of hormone-like compounds (Gravel et al., 2007; Contreras-Cornejo et al., 2009; Wang et al., 2020). Recent studies reveal that *Trichoderma* may activate the internal molecular mechanisms of plants, thereby altering hormonal signaling (Martínez-Medina et al., 2017; Patel et al., 2019; Wang et al., 2020). Wang et al. (2020) report that, in AUXR1, overexpressing poplar plantlets in which auxin is constitutively overproduced the level of indole-3-acetic acid decreases upon inoculation by *T. asperellum*.

The present results showed that the BCA Vintec^®^ alone negatively regulates auxin signaling and differentially regulates cytokinin signaling in grapevine trunk. The cross-talk between auxin and cytokinin signaling reportedly plays a major role in coordinating cell enlargement and cell division in meristem tissues (Aloni et al., 2006; Immanen et al., 2016). These studies show that auxin and cytokinin signaling act in an antagonistic but partially overlapping way (Schaller et al., 2015). Studies done on poplar provide insight into the distribution profiles of these two phytohormones during the activity of the vascular cambium, which is the lateral meristem where wood formation starts. Immanen et al. (2016) show that cell division in poplar vascular cambium increases significantly in transgenic trees, overexpressing a cytokinin biosynthesis-related gene from *Arabidopsis thaliana* (AtIP7). They also report that the levels of auxin and auxin-related genes also increase, which suggests that cytokinin functions as a major regulator of cambial activity.

During infection, no hormone-signaling-related functional category is enriched among genes up- or downregulated in plants pre-inoculated with Vintec^®^. On the contrary, the categories “Ethylene-signaling pathway” and “ABA biosynthesis” are enriched among DEGs in the presence of the two pathogens. Ethylene intervenes in many physiological processes; for instance, its cross-talk with auxin signaling regulates diverse plant developmental processes (Zemlyanskaya et al., 2018) and its interaction with jasmonic acid signaling modulates plant defense responses (Pieterse et al., 2012). Abscisic acid (ABA) is associated with abiotic stress responses, mainly drought and water stresses (Xie et al., 2005; Chen et al., 2020), but can play a role in the biotic stress response, which, in most cases, is considered to be a negative regulation of plant response (Mauch-Mani and Mauch, 2005; Asselbergh et al., 2008; Ueno et al., 2015). Various studies demonstrate that ABA interacts negatively with SA and JA/ET signaling (Anderson et al., 2004; Mauch-Mani and Mauch, 2005; Cao et al., 2011; Ueno et al., 2015). Altogether, this evidence combined with the present results suggests that both the pathogens and the BCA Vintec^®^ may interact with hormone signaling, altering hormone homeostasis in the trunk. Pathogens might use this mechanism to interfere with plant immunity, whereas the presence of Vintec^®^ could counteract this strategy.

Conversely, numerous studies report that *Trichoderma* spp. stimulate plant natural defenses before or during pathogen infection (Shoresh et al., 2010; Shaw et al., 2016). Mathys et al. (2012) show that T. *hamatum* T382 activates Induced Systemic Resistance (ISR) in *A. thaliana*, resulting in accelerated activation of the defense response to *Botrytis cinerea.* They postulated that the stimulation of the phenylpropanoid pathway by *T. hamatum* plays an essential role in ISR. Yan et al. (2021) also report that another species of *Trichoderma*, *T. harzianum*, enhances the accumulation of secondary metabolites such as phenols and flavonoids and suppresses root-knot nematode infections in tomato. Pratap Singh et al. (2021) also report a higher total phenolic content in plants pre-inoculated with *T. harzianum* and *T. asperellum* after *Sclerotinia sclerotium* infection in brinjal. The present study also reveals an enrichment in the secondary metabolism biosynthesis pathways during the infection, mostly associated with phenylpropanoids, flavonoids, and phenols.

Several studies highlight the accumulation of this type of metabolite in diseased grapevine wood (Amalfitano et al., 2000; Lima et al., 2012; Rusjan et al., 2017). Remarkably, the pattern of expression of genes associated with the biosynthesis of phenylpropanoids, flavonoids, or phenols depends on the presence or the absence of the BCA Vintec^®^ during the infection. Our results suggest that the presence of the BCA Vintec^®^ enhances the accumulation of stilbenes, which have antifungal properties, in accordance with parallel metabolomic work on the same pathosystem (Chervin et al., 2022). Stilbenes are described as phytoalexins in grapevine, so they are implicated in plant defense (Langcake and Pryce, 1977). For instance, the protective role of a number of stilbenes against *Plasmopara viticola*, the causal agent of downy mildew in grapevine, is described by Pezet et al. (2015) and Gabaston et al. (2017). In addition, a number of studies investigate how elicitation with chitosan affects grapevine gray mold (Iriti et al., 2004) and powdery mildew (Ait Barka et al., 2004; Romanazzi et al., 2021). The mechanism underlying the protection of *V. vinifera* by chitosan elicitation is related to the enhanced production of stilbenes in grapes (Lucini et al., 2018). Recently, a study comparing tolerant *V. vinifera* subspecies (*V. vinifera* subs. *Sylvestris*) and susceptible *V. vinifera* subs. *vinifera* showed that, after infection by *Neofusicoccum parvum*, tolerant subspecies contained more resveratrol and its derivates than susceptible subspecies (Labois et al., 2020). Although the role of these compounds in esca disease has not yet been completely elucidated, all this evidence suggests that stilbenes play a key role in the protection of grapevine against trunk diseases.

## Conclusion

This study shows that the application of the BCA Vintec^®^—a *Trichoderma atroviride* SC1 commercial solution—enhances modifications in the gene response to grapevine trunk diseases, both alone and during pathogen infection with two pathogens associated with esca disease. The BCA Vintec^®^ seems to alter in different ways the hormone homeostasis both alone and during infection with *P. minimum* and *P. chlamydospora*. Only during the infection does the BCA Vintec^®^ promote the expression of genes related to the biosynthesis of stilbenes, phenols, and flavonoids, which are metabolites known for their antifungal properties. Altogether, these results suggest that the BCA Vintec^®^ interferes in the plant response to esca-associated pathogens by enhancing the primary defense response.

## Data Availability Statement

The datasets presented in this study]can be found in online repositories. The name of the repository and accession number can be found below: NCBI; PRJNA817518.

## Author Contributions

AR-O and AJ conceived the experimental plan. AJ coordinated the study. AR-O conducted the experiments (plant production, RNA and DNA extractions, qRT-PCR, and qPCR reactions), conducted RNA sequencing bioinformatics analysis, and wrote the manuscript. CB assisted with plant production and qRT-PCR reactions. AJ, TL, JC, and JD contributed to the data analysis and interpretation. All co-authors revised and contributed modifications to the final manuscript.

## Conflict of Interest

The authors declare that the research was conducted in the absence of any commercial or financial relationships that could be construed as a potential conflict of interest.

## Publisher’s Note

All claims expressed in this article are solely those of the authors and do not necessarily represent those of their affiliated organizations, or those of the publisher, the editors and the reviewers. Any product that may be evaluated in this article, or claim that may be made by its manufacturer, is not guaranteed or endorsed by the publisher.

## Footnotes

1
https://github.com/PF2-pasteur-fr/SARTools
