# Supplementary material for: Comparative Transcriptomics Suggests Early Modifications by Vintec® in Grapevine Trunk of Hormonal Signaling and Secondary Metabolism Biosynthesis in Response to Phaeomoniella chlamydospora and Phaeoacremonium minimum
Source: Front Microbiol. 2022 May 17;13:898356. doi: 10.3389/fmicb.2022.898356 (PMC9152730; doi:10.3389/fmicb.2022.898356)
Supplement: Supplementary file 1 [file Table_1.docx]

**Supplementary materials**

**Supplementary Table 1**. Number of total reads and percentage of reads aligned to the genome of *V. vinifera* by condition.

|  | **Input reads** | **Uniquely mapped reads** | | | **Mismatch rate** | **Multi-mapping reads** | | | **Unmapped reads** | | |
| --- | --- | --- | --- | --- | --- | --- | --- | --- | --- | --- | --- |
|  |  | **Single** | **Double** | **%** | **per base %** | **Single** | **Double** | **%** | **Single** | **Double** | **%** |
| NINi_1 | 24843544 | 8900000 | 17800000 | 69,9 | 0,88 | 459987 | 919974 | 3,70 | 1122086 | 2244172 | 9,03 |
| NINi_2 | 22494300 | 7200000 | 14400000 | 70,3 | 0,9 | 367939 | 735878 | 3,27 | 949257 | 1898514 | 8,44 |
| NINi_3 | 19995758 | 8479130 | 16958260 | 84,81 | 0,55 | 136442 | 272884 | 1,36 | 1381857 | 2763714 | 13,82 |
| NINi_4 | 24402280 | 8300000 | 16600000 | 72 | 0,88 | 464441 | 928882 | 3,81 | 1127031 | 2254062 | 9,24 |
| INi_1 | 18735586 | 8102252 | 16204504 | 86,49 | 0,5 | 137860 | 275720 | 1,47 | 1128173 | 2256346 | 12,04 |
| INi_2 | 18857856 | 8189218 | 16378436 | 86,85 | 0,54 | 138084 | 276168 | 1,46 | 1102067 | 2204134 | 11,69 |
| INi_3 | 25056288 | 7000000 | 14000000 | 72,8 | 0,9 | 462212 | 924424 | 3,69 | 1078765 | 2157530 | 8,61 |
| INi_4 | 20466128 | 8500000 | 17000000 | 66,1 | 0,9 | 438584 | 877168 | 4,29 | 1004563 | 2009126 | 9,82 |
| IV_1 | 16940134 | 7430883 | 14861766 | 87,73 | 0,53 | 128509 | 257018 | 1,52 | 910675 | 1821350 | 10,75 |
| IV_2 | 18802204 | 8192313 | 16384626 | 87,14 | 0,54 | 141790 | 283580 | 1,51 | 1066999 | 2133998 | 11,35 |
| IV_3 | 18417968 | 8017479 | 16034958 | 87,06 | 0,55 | 140145 | 280290 | 1,52 | 1051360 | 2102720 | 11,42 |
| IV_4 | 16965500 | 7416999 | 14833998 | 87,44 | 0,53 | 129889 | 259778 | 1,53 | 935862 | 1871724 | 11,03 |
| IPP_1 | 17948418 | 8974209 | 17948418 | 85,5 | 0,54 | 135570 | 271140 | 1,51 | 1166006 | 2332012 | 12,99 |
| IPP_2 | 16376082 | 7065320 | 14130640 | 86,29 | 0,55 | 123908 | 247816 | 1,51 | 998813 | 1997626 | 12,20 |
| IPP_3 | 21552894 | 9090975 | 18181950 | 84,36 | 0,56 | 160413 | 320826 | 1,49 | 1525059 | 3050118 | 14,15 |
| IPP_4 | 24276936 | 10317401 | 20634802 | 85 | 0,56 | 174434 | 348868 | 1,44 | 1646633 | 3293266 | 13,57 |
| IVPP_1 | 22628288 | 9630876 | 19261752 | 85,12 | 0,57 | 168249 | 336498 | 1,49 | 1515019 | 3030038 | 13,39 |
| IVPP_2 | 18815794 | 7791688 | 15583376 | 82,82 | 0,57 | 132482 | 264964 | 1,41 | 1483727 | 2967454 | 15,77 |
| IVPP_3 | 17467546 | 7510473 | 15020946 | 85,99 | 0,55 | 121343 | 242686 | 1,39 | 1101957 | 2203914 | 12,62 |
| IVPP_4 | 23214594 | 9994696 | 19989392 | 86,11 | 0,57 | 171174 | 342348 | 1,47 | 1441427 | 2882854 | 12,42 |
| Average | 20412905 |  |  |  |  |  |  |  |  |  |  |
| Standard deviation | 2901446 |  |  |  |  |  |  |  |  |  |  |
