# Supplementary figures and images for: Comparative Transcriptomics Suggests Early Modifications by Vintec® in Grapevine Trunk of Hormonal Signaling and Secondary Metabolism Biosynthesis in Response to Phaeomoniella chlamydospora and Phaeoacremonium minimum
Source: Front Microbiol. 2022 May 17;13:898356. doi: 10.3389/fmicb.2022.898356 (PMC9152730; doi:10.3389/fmicb.2022.898356)

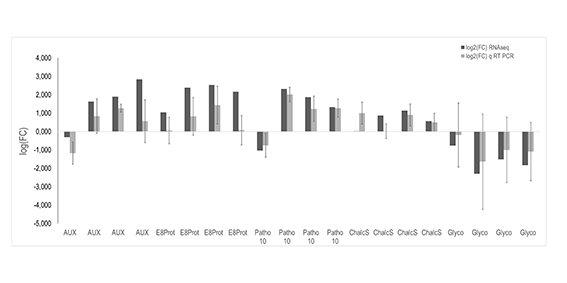

Supplement: Supplementary file 2 [file Image_1.TIF]

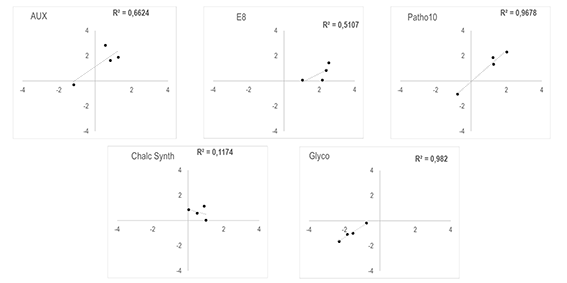

Supplement: Supplementary file 3 [file Image_2.TIF]
